# *In Vitro* Sustained Release Study of Gallic Acid Coated with Magnetite-PEG and Magnetite-PVA for Drug Delivery System

**DOI:** 10.1155/2014/416354

**Published:** 2014-03-05

**Authors:** Dena Dorniani, Aminu Umar Kura, Samer Hasan Hussein-Al-Ali, Mohd Zobir Bin Hussein, Sharida Fakurazi, Abdul Halim Shaari, Zalinah Ahmad

**Affiliations:** ^1^Materials Synthesis and Characterization Laboratory (MSCL), Institute of Advanced Technology (ITMA), Universiti Putra Malaysia, 43400, Selangor, Malaysia; ^2^Vaccines and Immunotherapeutics Laboratory (IBS), Universiti Putra Malaysia, 43400, Selangor, Malaysia; ^3^Laboratory of Molecular Biomedicine, Institute of Bioscience, Universiti Putra Malaysia, 43400, Selangor, Malaysia; ^4^Physics Department, Faculty of Science, Universiti Putra Malaysia, 43400, Selangor, Malaysia; ^5^Chemical Pathology Unit, Department of Pathology, Faculty of Medicine and Health Sciences, Universiti Putra Malaysia, 43400, Selangor, Malaysia

## Abstract

The efficacy of two nanocarriers polyethylene glycol and polyvinyl alcohol magnetic nanoparticles coated with gallic acid (GA) was accomplished via X-ray diffraction, infrared spectroscopy, magnetic measurements, thermal analysis, and TEM. X-ray diffraction and TEM results showed that Fe_3_O_4_ nanoparticles were pure iron oxide having spherical shape with the average diameter of 9 nm, compared with 31 nm and 35 nm after coating with polyethylene glycol-GA (FPEGG) and polyvinyl alcohol-GA (FPVAG), respectively. Thermogravimetric analyses proved that after coating the thermal stability was markedly enhanced. Magnetic measurements and Fourier transform infrared (FTIR) revealed that superparamagnetic iron oxide nanoparticles could be successfully coated with two polymers (PEG and PVA) and gallic acid as an active drug. Release behavior of gallic acid from two nanocomposites showed that FPEGG and FPVAG nanocomposites were found to be sustained and governed by pseudo-second-order kinetics. Anticancer activity of the two nanocomposites shows that the FPEGG demonstrated higher anticancer effect on the breast cancer cell lines in almost all concentrations tested compared to FPVAG.

## 1. Introduction

Recently, nanoparticles are attracting considerable attention in biomedical applications due to their superior physical and chemical properties. In biomedical applications which required core-shell magnetic nanoparticles, a metal or metallic oxide core, encapsulated in a polymeric coating, resulted in stable, biocompatible, and biodegradable nanoparticles. For superparamagnetic iron oxide nanoparticles, Fe is being reused/recycled by cells using normal biochemical pathways for the Fe metabolism [1–3]. In drug delivery system, superparamagnetism is essential because, whenever the external magnetic field [4] is removed, magnetization disappears, and therefore agglomeration in capillary vessels can be avoided [5].

If the Fe-based magnetic materials consisting of very small crystallites, saturation magnetization is found to decrease sharply which is related to crystalline magnetic anisotropy constant and nanoparticles become superparamagnetic at sizes <25 nm [2, 6, 7]. Polymeric nanoparticles have superior ability to target drugs and reducing toxic side effects on healthy cells and tissues. Polymeric nanoparticles are colloidal solid with spherical, branched, or shell structures with various sizes ranging from 10 to 1000 nm [8]. Due to the coating of nanoparticles with a neutral and hydrophilic compound such as polyethylene glycol (PEG) [9, 10], polyvinyl alcohol (PVA) [11, 12], polysaccharides [13], and dysopsonins (HSA), the circulatory half-life can be increased from minutes to hours or days. Polyvinyl alcohol (PVA) is a hydrophilic polymer with a simple chemical structure: high hydroxyl group which is suitable for biomedical applications due to desired many properties such as biocompatibility, nontoxicity, noncarcinogenicity, nonimmunogenicity, and inertness in body fluids. Due to promising biomaterial properties, several studies have focused on the application of PVA in biomedical and pharmaceutical fields.

Drugs can be adsorbed, dissolved, entrapped, attached, or encapsulated into the nanoparticles matrix and resulted in the nanoparticles with sustained release of drugs over longer time periods [8, 14]. Gallic acid (3,4,5-trihydroxybenzoic acid), an anticancer drug, can be obtained from a variety of natural products such as gallnut, sumac, and black tea [15–17]. Apart from anticarcinogenic properties it also has antimutagenic, antiviral, anti-inflammatory, and antimicrobial agent properties [15, 17–19].

This study concerns the comparing of the immobilization of gallate anion on the surface of magnetite nanoparticles preprepared using polyethylene glycol (PEG) and polyvinyl alcohol (PVA) as a polymer stabilizer, to improve the reducing of the size distribution of the nanoparticles and active delivery to specific cells targeting in normal human fibroblasts (3T3) and in several cancer cell lines. In this study, magnetite was chosen as a core and gallate anion-PVA or PEG was chosen as shells to be adsorbed on the surface of the core. In this paper, results from the XRD, FTIR, magnetite studies, TGA/DTG, particle size analysis, and cytotoxicity as well as release property of gallate anion from both nanocomposites into aqueous media will be discussed.

## 2. Materials and Methods

### 2.1. Materials

Distilled deionized water (18.2 M·Ωcm^−1^) was used in all experiments. Iron (II) chloride tetrahydrate (FeCl_2_·4H_2_O ≥ 99%), iron (III) chloride hexahydrate (FeCl_3_·6H_2_O, 99%), and polyvinyl alcohol (98% degree of hydrolysis) were purchased from Merck, Germany. Polyethylene glycol, average M.W. 300, was purchased as a raw material from Acros Organics BVBA. Ammonia solution (25%) was obtained from Scharlau, and gallic acid with 97% purity was supplied by Sigma-Aldrich (St. Louis, MO).

### 2.2. Preparation of Magnetite Nanoparticles

Iron oxide nanoparticles were prepared as previously reported by Lee et al. [20]. In order to prepare magnetite iron oxide coated with polyethylene glycol and gallic acid (FPEGG), the mixture of 2.43 g ferrous chloride tetrahydrate (FeCl_2_·4H_2_O), 0.99 g ferric chloride hexahydrate (FeCl_3_·6H_2_O), and 80 mL deionized water in the presence of 6 mL ammonia hydroxide (25% by mass) was exposed to ultrasonic irradiation for 1 h. The precipitates were centrifuged and washed 3 times and then the washed precipitates were dispersed in 100 mL deionized water and mixed with 1% PEG. After stirring the mixture for 24 hours, the black precipitates were collected by a permanent magnet and washed three times to remove the excess PEG which does not participate in the coating process and then dried in an oven. The 2% of drug, GA [4, 21] which was dissolved in deionized water, was added into the magnetite-PEG and the mixture was stirred for 24 h. Finally, the coated magnetite was washed and dried in an oven. The same procedure was done to prepare magnetite iron oxide coated with polyvinyl alcohol-gallic acid.

## 3. Cell Viability Study

### 3.1. Cell Culture

Normal lung's cells and breast cancer cell lines were obtained from the American Tissue Culture Collection (VA, USA); DMEM/F12 (Dulbecco's modified essential medium/Ham's 12 nutrient mixture, Gibco), supplemented with 10% (v/v) foetal bovine serum (JS Bioscience, Australia), and 1% (v/v) antibiotic (2 mML glutamine, 100 U/mL penicillin, and 0.1 mg/mL streptomycin; Gibco) were used to culture the cell lines. A temperature controlled (37°C) and 5% humidified CO_2_ incubator was used to keep the cells. Enzymatically detached cells (trypsin) after confluence were resuspended in new media and seeded in a 96-well plate for treatment and onward viability assays and in both cell lines 90% confluence was used before seeding.

### 3.2. Preparation of FNPs, FPEGG, and FPVAG Nanocomposites for Viability Assay

Experiments were done in triplicate and each time freshly prepared FNPs and both nanocomposites (FPEGG and FPVAG) were used to treat cells. In order to ensure the uniform suspension, stock suspensions of 10 mg/mL of each nanocomposites and FNPs were made by sonication for 30 minutes and culture medium was used to obtain the desired concentration via serial dilution. The nanoparticles coated with gallic acid-PVA (FPVAG) and gallic acid-PEG (FPEGG) as well as the corresponding iron oxide nanoparticles (FNPs) without any coating were initially dispersed into the phosphate buffered solution. To further disperse the FNPs and both nanocomposites, vortex agitation for 2 min was used prior to treatment. Dose range of 0.78 **μ**g/mL to 25.00 **μ**g/mL was used, and cells were exposed for 72 hr to assess the impact of the treatment on the cells viability; wells containing cells and media only were used as control for comparison.

### 3.3. Cytotoxicity Testing

Cytotoxicity testing was performed using MTT assay. Principally only viable cells through their mitochondrial dehydrogenase enzymes reduce the tetrazolium salt to form a blue formazan product [22]. Cells were seeded in 96-well plates (Nunc, Denmark) at a density of 1∗10^5^ cells/mL in 100 **μ**L medium containing 10% FBS. The principle of this assay is by reduction of the tetrazolium salt by the mitochondrial dehydrogenase of only viable cells, which forms a blue formazan product. Twenty-four-hour period was chosen to allow cells to adhere in the 96-well plates and cells were treated with the selected increasing concentrations of each nanomaterial prepared as well as pure gallic acid. Control and test exposure media were removed after 72 hr of exposure and each well was rinsed with PBS. To each well 100 **μ**L of new media containing 10% MTT solution was added and incubated in an incubator for 4 hr allowing yellow formazan formation by the viable cells (MTT solution is made as 5 mg/mL PBS). After the 4 hr incubation period, the medium was discarded, the cells were washed with 100 **μ**L of PBS, and 100 **μ**L of DMSO was added to each well to extract the dye. Absorbance was measured at 570 nm and a background absorbance was measured at 630 nm. Then cytotoxicity was calculated as Average of treated/Average of control ∗ 100%.

### 3.4. Controlled-Release Procedure

In order to evaluate the drug release profiles of gallic acid from FPEGG and FPVAG, two pH values (7.4 and 4.8) were used at 25°C [23–26]. The release of GA was achieved by adding 10 mg of FPEGG and FPVAG nanocomposites into the mixture of 1 mL HCl and 3 mL HNO_3_ and marked them up to 10 mL by deionized water after stirring for 24 hours. Different anions such as Cl^−^, HPO_4_^2−^, and H2PO_4_^−^ were used in this study. Using ultraviolet-visible spectroscopy, the accumulated amount of GA released from FPEGG and FPVAG nanocomposites was measured at *λ*_max⁡_ = 264 nm.

### 3.5. Characterization

Powder X-ray diffraction (PXRD) was recorded using a Shimadzu diffractometer XRD-6000 (Tokyo, Japan) instrument to determine the crystal structure of the samples in a range of 6–70° using CuK_*α*_ radiation (*λ* = 1.5406 Å) at 40 kV and 30 mA. Fourier transform infrared (FTIR) spectra of the materials were obtained over the range of 400–4000 cm^−1^ on a Thermo Nicolet Nexus FTIR (model smart orbit) with 4 cm^−1^ resolution, using a KBr disc method with approximately 1% of the sample in 200 mg of spectroscopic grade potassium bromide, and the pellets were pressed at 10 tons. Thermogravimetry analysis (TGA) and differential thermogravimetry analysis (DTG) were performed using a Mettler-Toledo Instrument (Longview, WA) in 150 **μ**L alumina crucibles in the range of 20–1000°C at a heating rate of 10 degrees per minute. Transmission electron microscopy (TEM) was used to observe the mean particle size, size distribution, and morphology of the samples using a Hitachi H-7100 at an accelerating voltage of 100 kV for iron oxide nanoparticles and FPEGG nanocomposite and 80 kV for FPVAG nanocomposite. To observe the optical properties and a controlled-release study of GA from FPVAG and FPEGG nanocomposites, ultraviolet-visible spectra (Shimadzu 1650 Series, Tokyo, Japan) were used.

## 4. Results and Discussion

### 4.1. X-Ray Diffraction

**Figure 1:**
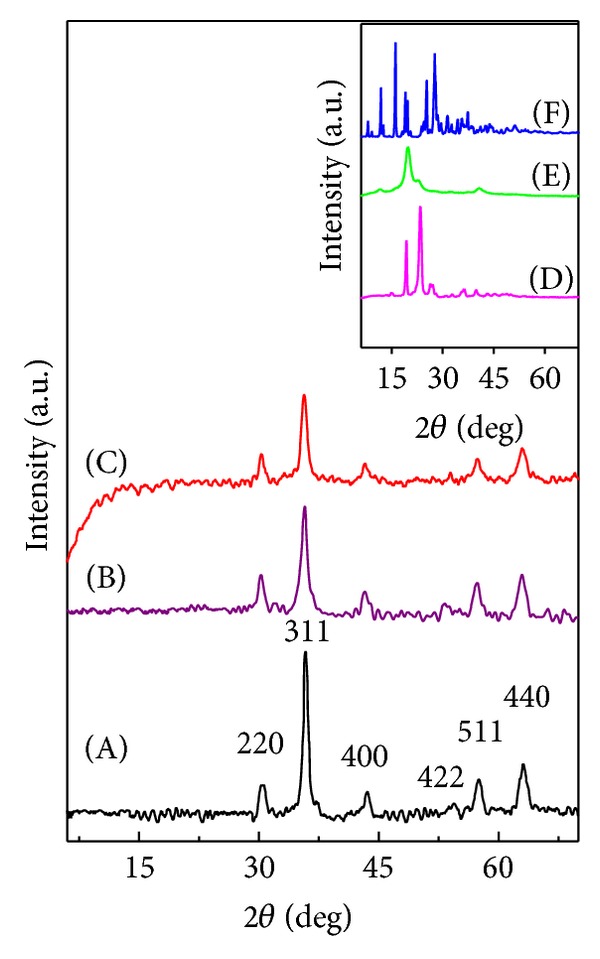
XRD patterns of FNPs (A), FPVAG (B), and FPEGG (C). The inset shows the XRD patterns of pure PEG (D), pure PVA (E), and pure GA (F). FPEGG: iron oxide coated with polyethylene glycol and gallic acid, FPVAG: iron oxide coated with polyvinyl alcohol and gallic acid, PEG: pure polyethylene glycol, PVA: pure polyvinyl alcohol, and GA: pure drug gallic acid.

Representative powder X-ray diffraction patterns of bare iron oxide nanoparticles, iron oxide nanoparticles coated with PVA-gallic acid (FPVAG), and iron oxide nanoparticles coated with PEG-gallic acid (FPEGG) are presented in Figure 1. The inset in Figures 1(D), 1(E), and 1(F) shows the X-ray diffraction spectrum of pure PEG, PVA, and GA, respectively. Figure 1(D) (pure PEG) shows two main diffraction peaks with high intensity at 2*θ* = 19.3° and 23.5°. The broad peak pertaining to PVA at 2*θ* = 19.5° can be due to the amorphous nature of the PVA (Figure 1(E)). All nanocomposites have six characteristic peaks at 2*θ* = 30.16°, 35.95°, 43.34°, 54.17°, 57.27° and 62.98°, which can be indexed to the (220), (311), (400), (422), (511), and (440) Bragg reflection, respectively. From the XRD analysis, it has also been found that these six diffraction peaks correspond to the pure magnetite nanoparticles with a cubic inverse spinal structure (Reference JCPDS Number 82-1533). Because of the lack of the characteristic superlattice diffractions at (210), (213), and (300), it can be found that the maghemite (Fe_2_O_3_, *γ*-Fe_2_O_3_) does not exist in the as-synthesized iron oxide and both nanocomposites [27, 28]. Owing to observing these characteristic peaks in all three nanocomposites (Figures 1(A), 1(B), and 1(C)), it is evident that the coating process did not result in a phase change of the iron oxide nanoparticles.

From the Debye-Scherrer formula (*D* = *Kλ*/*β*cos⁡*θ*), the average crystallite size has been calculated for the bare iron oxide nanoparticles. The mean crystallite size of pure iron oxide nanoparticles was about 3 nm.

### 4.2. Infrared Spectroscopy (FTIR)

**Figure 2:**
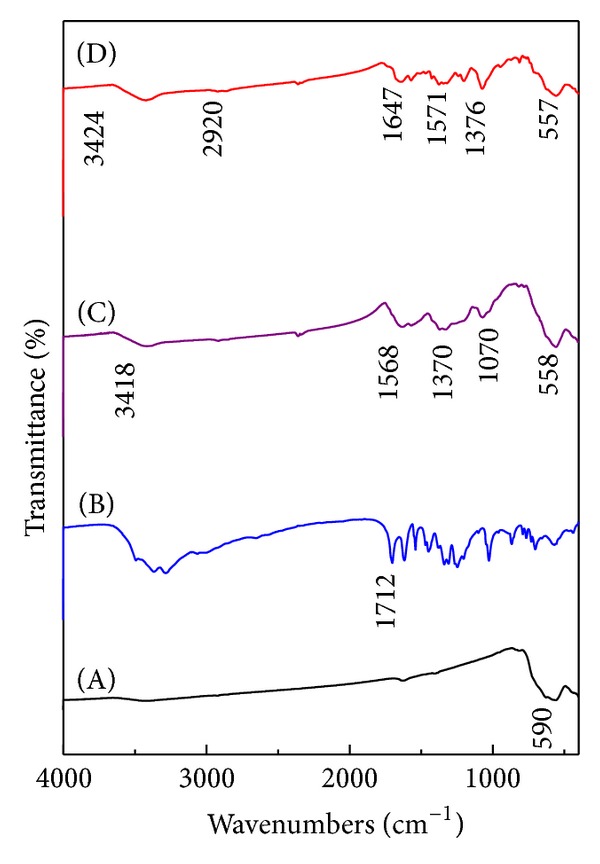
FTIR spectra of FNPs (A), pure GA (B), FPVAG (C), and FPEGG nanocomposite (D).

Figures 2(A)–2(D) show the FTIR spectra of FNPs, GA, FPVAG, and FPEGG, respectively.  Figure 2(A) shows that the magnetite iron oxide nanoparticles (FNPs) have absorption peak at 590 cm^−1^, which is due to Fe–O stretching in Fe_3_O_4_. However, the FPVAG and FPEGG nanocomposites show characteristic peaks of Fe–O at 558 and 557 cm^−1^, respectively, which confirm the presence of magnetite nanoparticles in both nanocomposites.

In FPVAG nanocomposite (Figure 2(C)), the alcoholic O–H stretching band was observed at 3418 cm^−1^. In addition, a band at 2917 cm^−1^ is corresponding to C–H stretching vibration, and 1070 cm^−1^ is attributable to M–O–C (M=Fe) bond [28]. This evidence confirms the attachment of PVA onto iron oxide nanoparticles via hydrogen bond between hydroxyl group of PVA and protonated surface of the oxide [28]. FTIR spectrum of the FPVAG nanocomposite shows the characteristic peaks for GA, confirming that the loaded drug on the surface of PVA is GA, for example, the peaks observed at 1568 and 1370 cm^−1^, which are due to asymmetry and symmetry COO^−^ stretching, respectively.

In FPEGG nanocomposite (Figure 2(D)), the presence of hydroxyl groups formed to link PEG to the oxide surface was confirmed by the absorbance peaks for –OH stretching at 3424 cm^−1^ and –OH out-of-plane bending vibration at 529 cm^−1^ on the nanocomposite FTIR spectra [29]. In addition, the appearance of peaks at 2920 and 947 cm^−1^ for –CH_2_ stretching vibration and –CH out-of-plane bending vibration, respectively, confirms the presence of PEG on the nanoparticle surface [30]. FTIR spectrum of the FPEGG nanocomposite shows the characteristic peaks for GA, confirming that the drug loaded on the surface of PEG is GA. For instance, the peaks at 1462 and 1246 cm^−1^ are due to the –OH stretching [30], and the peaks observed at 1571 and 1376 cm^−1^ are due to asymmetry and symmetry COO^−^ stretching, respectively.

### 4.3. Thermogravimetric Analysis

**Figure 3:**
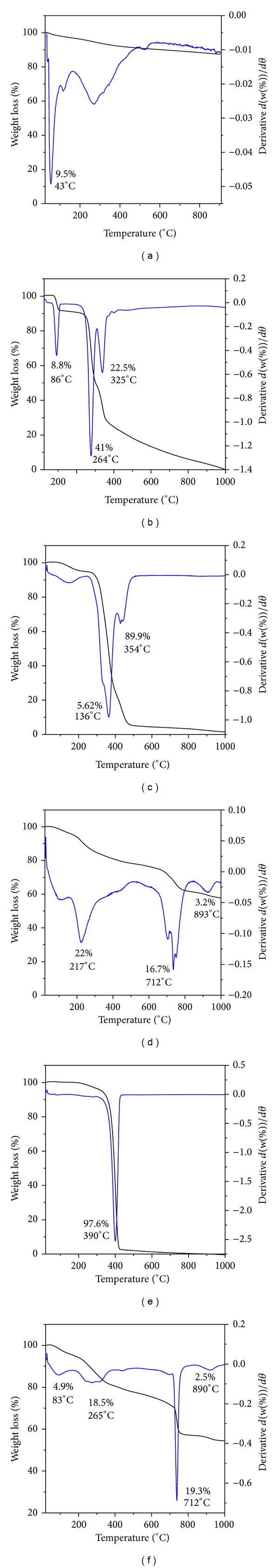
TGA/DTG of (a) FNPs, (b) GA, (c) PVA, (d) FPVAG nanocomposite, (e) pure PEG, and (f) FPEGG nanocomposite.

The thermogravimetric and differential thermogravimetric analyses obtained for FNPs, GA, PVA, FPVAG, PEG, and FPEGG are shown in Figure 3, respectively. The TGA curves of FNPs (Figure 3(a)) show that the weight loss over the temperature range from 25°C to 1000°C was about 9.5%. This might be due to the loss of residual water in the sample. For PVA polymer (Figure 3(c)), two main thermal events were clearly observed. The first event occurred in the region of 50–235°C with 5.6% weight loss. This was followed by the second stage at 235–509°C, with 89.9% weight loss. Comparing the TGA curve of FNPs, PVA with FPVAG shows the curve indicating the presence of GA in the final nanocomposite.  Figure 3(e) shows that PEG polymer has only one-stage weight loss, in the region of 170–433°C with 97.6% weight loss. The FPEGG nanocomposite shows weight loss starting from 36°C and completed at 961°C with four weight losses (36–161°C, 4.9%; 171–543°C, 18.5%; 549–793°C, 19.3%; and finally 811–961°C, 2.5%). The range of weight loss in the nanocomposite is higher than in the PEG, indicating the presence of GA in the final nanocomposite.

### 4.4. Magnetic Properties

**Figure 4:**
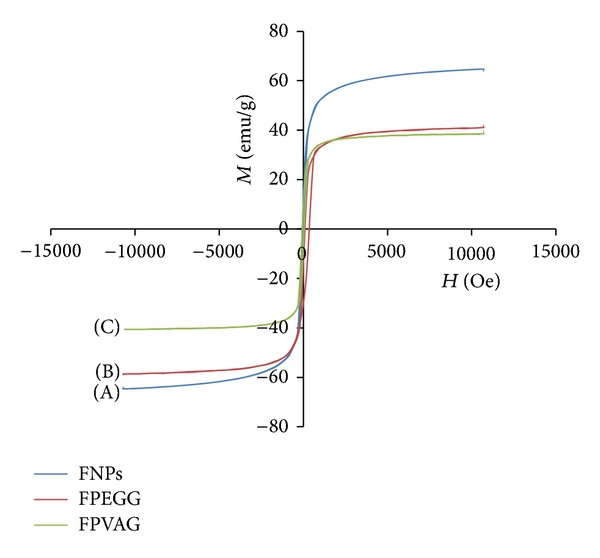
Magnetization plots of (A) FNPs, (B) FPEGG, and (C) FPVAG.

Superparamagnetism is playing a key role for magnetic targeting carriers and biomedical applications and the lack of hysteresis is one of the criteria to identify the product as superparamagnetic [31]. The most important parameters in vibrating sample magnetometers (VSM) extracted from hysteresis loops are saturation magnetization (Ms), remanence magnetization (Mr), and the coercivity (Hc). The value of Ms (magnetization at maximum applied field) can be enhanced with the increases of crystallinity. Coercivity (Hc) is the field required for demagnetizing the sample and the low shape magnetic anisotropy can cause the lower value of Hc and Ms. Superparamagnetic materials have high saturation magnetization and zero coercivity and remanence magnetization (the magnetization at zero applied field after applying a saturation field).  Figure 4 shows the hysteresis loops of naked iron oxide nanoparticles (Figure 4(A)), iron oxide coated with polyethylene glycol-gallic acid (FPEGG) (Figure 4(B)), and iron oxide coated with polyvinyl alcohol-gallic acid (FPVAG) characterized by vibrating sample magnetometer (VSM) at room temperature. The saturation magnetization of magnetite nanoparticles synthesized by coprecipitation method was about 64.65 emu/g compared to 40.00 emu/g and 38.63 emu/g for FPEGG and FPVAG nanocomposite, respectively, which agrees nicely with previous works [28, 32, 33]. It is clear that the saturation magnetization of bare iron oxide nanoparticles depends on the method of synthesis as well as the size of the nanoparticles [34].

Therefore, the amount of saturation magnetization is usually lower than the theoretical value owing to surface inhomogeneities [2, 35, 36]. The remanence magnetization value for naked iron oxide was about 1.57 emu/g compared to 1.13 emu/g and 0.84 emu/g after coating with polyethylene glycol-gallic acid and polyvinyl alcohol-gallic acid, respectively. This observation also could be due to the fact that the nanoparticles which are covered were so small that they might be assumed to have a single magnetic domain [37].

Saturation magnetization of the prepared magnetic iron oxide nanoparticles, FPEGG and FPVAG nanocomposites, was high; however the remanent magnetization and coercivity (Hc) were low. This demonstrates that all samples are soft superparamagnetic; that is, after removal of a magnetic field they did not retain any magnetism reducing the probability of particle aggregation because of magnetic dipole attraction [38, 39]. The decrease of the saturation magnetization after coating with PEG-GA and PVA-GA is only due to the existence of coated materials on the surface of magnetite nanoparticles [40]. The values of saturation magnetization (Ms), remanent magnetization (Mr), and coercivity (Hc) are shown in Table 1.

### 4.5. Determination of Average Particle Size and Particle Size Distribution

**Figure 5:**
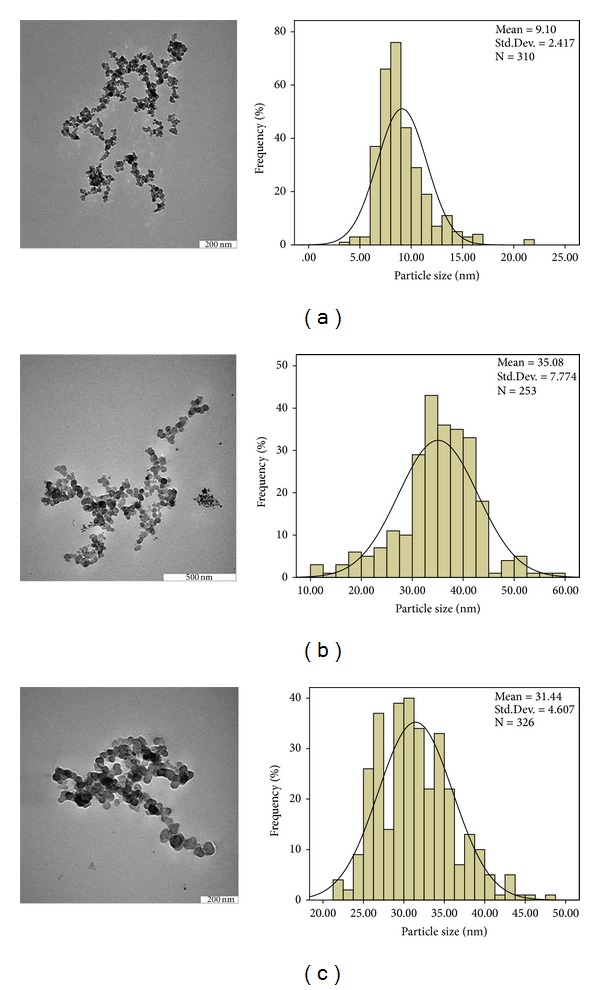
TEM micrographs of (a) FNP with 200 nm microbar and the particle size distribution, (b) FPVAG with 500 nm microbar and its particle size distribution, and (c) FPEGG nanocomposite with 200 nm microbar and the particle size distribution.

The typical transmission electron micrographs (TEM) and size distribution of as-prepared FNPs, FPVAG, and FPEGG nanocomposites are shown in Figure 5. The obtained images showed that the particles with nanometer size were successfully prepared by coprecipitation method and were essentially monodisperse (Figures 5(a), 5(b), and 5(c)). It was clear that FNPs and both the nanocomposites display roughly spherical shapes. The particle size and size distribution of the iron oxide nanoparticles, FPVAG and FPEGG nanocomposite, were determined by measuring around 300 particles randomly using image analysis software (a UTHSCSA Image Tool). From these images it was obvious that the particles had a very small size range between 9 and 35 nm in diameter with a narrow size distribution. The average diameter of the bare FNPs is 9 ± 2 nm, whereas after coating with PVA-GA and PEG-GA the mean size of FPVAG and FPEGG nanocomposites increased to 35 ± 7 and 31 ± 4, respectively. The results showed that there are no too many differences between the sizes of FPEGG and FPVAG nanocomposites. The enlargement of the size of FPVAG and FPEGG after coating procedure can be used to prove the formation of iron oxide nanoparticles coated with PVA-GA and PEG-GA [41].

### 4.6. Loading and Release Behavior of Gallic Acid

**Figure 6:**
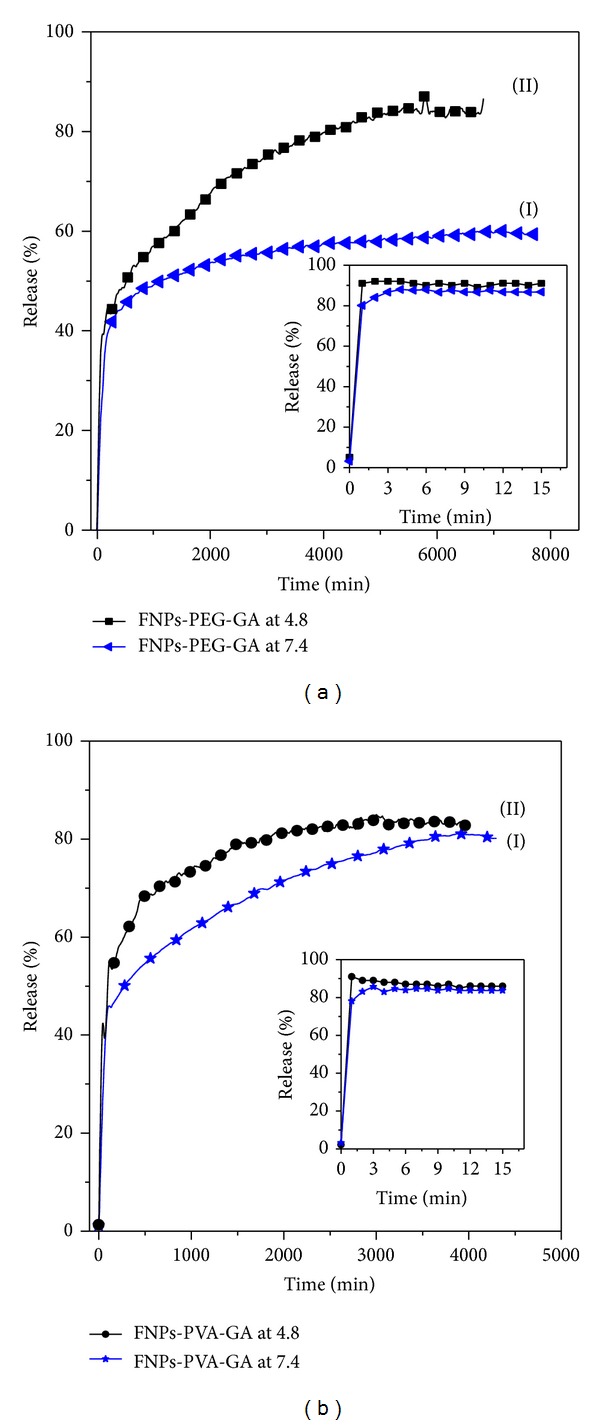
(a) Release profiles of GA from the FPEGG nanocomposite into (I) phosphate buffered solution at pH 7.4 and (II) phosphate buffered solution at pH 4.8 and (b) release profiles of GA from the FPVAG nanocomposite into (I) phosphate buffered solution at pH 7.4 and (II) phosphate buffered solution at pH 4.8. Note: inset in (a) shows the release profiles of GA from its physical mixture of FNPs-PEG-GA at pH 7.4 and 4.8, and inset in (b) shows the release profiles of GA from its physical mixture of FNPs-PVA-GA at pH 7.4 and 4.8.

The percentages of loading of gallic acid in FPEGG and FPVAG were investigated via the ultraviolet-visible absorption spectroscopy. Loading of GA into FPVAG was found to be around 5% compared to 7% for the FPEGG nanocomposite. The release profiles for GA from the two mentioned nanocomposites were investigated in phosphate buffered solutions at pH 7.4 and 4.8 (Figure 6).

The release profiles of GA from FPEGG show that the maximum percentage release reaches about 60.9% within about 6905 min (115 h) at pH 7.4 compared to 89.4% within about 5775 min when exposed to pH 4.8 (Figure 6(a)). The inset of Figure 6(a) shows the physical mixture of GA and FNPs-PEG exposed to either pH 4.8 or pH 7.4. It was found that GA was quickly released from the physical mixture of FNPs-PEG-GA and that release was complete within 2 and 4 minutes at pH 4.8 and pH 7.4, respectively. In addition, the release profiles of GA from FPVAG reveals that the maximum percentage release reaches about 80.9% within about 6594 min at pH 7.4 compared to 86.4% within about 3045 when exposed to buffered solution at pH 4.8 (Figure 6(b)). Also the physical mixture of GA with FNPs-PVA into phosphate buffered solution at pH 4.8 and pH 7.4 shows the rapid release during the initial few minutes (inset of Figure 6(b)). The physical mixture of GA and both FNPs-PVA and FNPs-PEG showed no sustained-release effects in both phosphate buffered solutions at pH 4.8 and pH 7.4 due to low electrostatic attraction between the GA anions and both FNPs-PVA and FNPs-PEG, respectively. From the results, it was found that the release profile of GA from FPEGG was more sustained compared to FPVAG nanocomposite.

### 4.7. Release Kinetics of Gallic Acid from the Nanocomposites FPEGG and FPVAG

**Figure 7:**
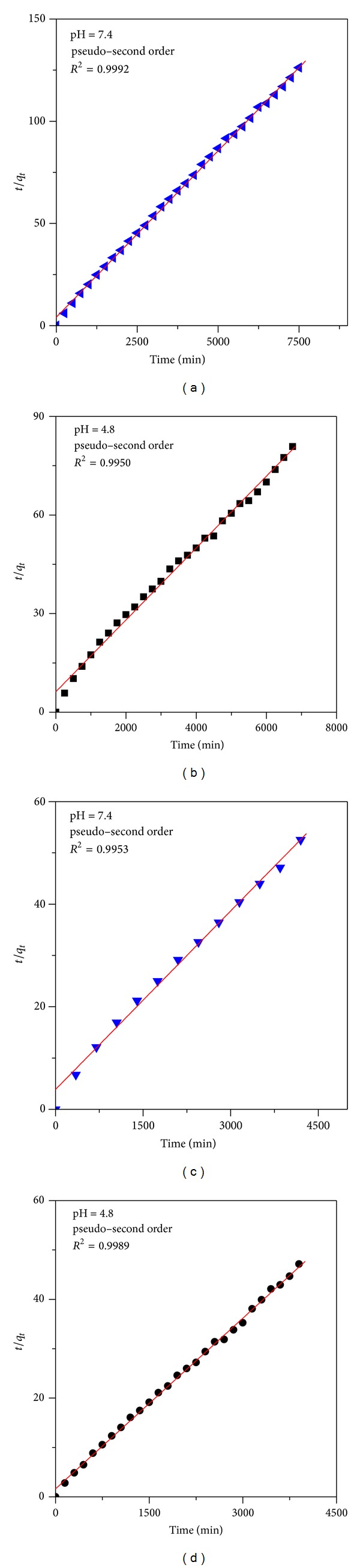
Fitting the data of GA release from FPEGG into different solutions to the pseudo-second-order kinetics for pH 7.4 (a) and pH 4.8 (b) and fitting data of GA released from FPVAG into different solutions to the pseudo-second-order kinetics for pH 7.4 (c) and pH 4.8 (d).

The release kinetics behavior of gallic acid from FPEGG and FPVA nanocomposite could be investigated by different kinetics models such as first-order [42] (ln⁡(*q*_*e*_ − *q*_*t*_) = ln⁡*q*_*e*_ − *k*_1_ *t*), pseudo-second-order [43] (*t*/*q*_*t*_ = 1/*k*_2_ *q*_*e*_^2^ + *t*/*q*_*e*_), and parabolic diffusion [44] (1 − *M*_*t*_/*M*_0_)/*t* = *kt* ^−0.5^ + *b*) equations. For the above equations, the *q*_*e*_ and *q*_*t*_ are the equilibrium release rate and the release rate at time *t*, respectively; *k* is a constant corresponding to release amount; and *M*_0_ and *M*_*t*_ are the drug content remaining in FPEGG and FPVAG nanocomposites at release times 0 and *t*, respectively. On the basis of these models, mentioned earlier, it was found that the pseudo-second-order kinetic model can be better fitted to describe the release behavior of gallic acid from FPEGG and FPVAG nanocomposites compared to the other models used in this study (Figures 7(a), 7(b), 7(c), and 7(d) and Table 2).

### 4.8. *In Vitro* Bioassay

**Figure 8:**
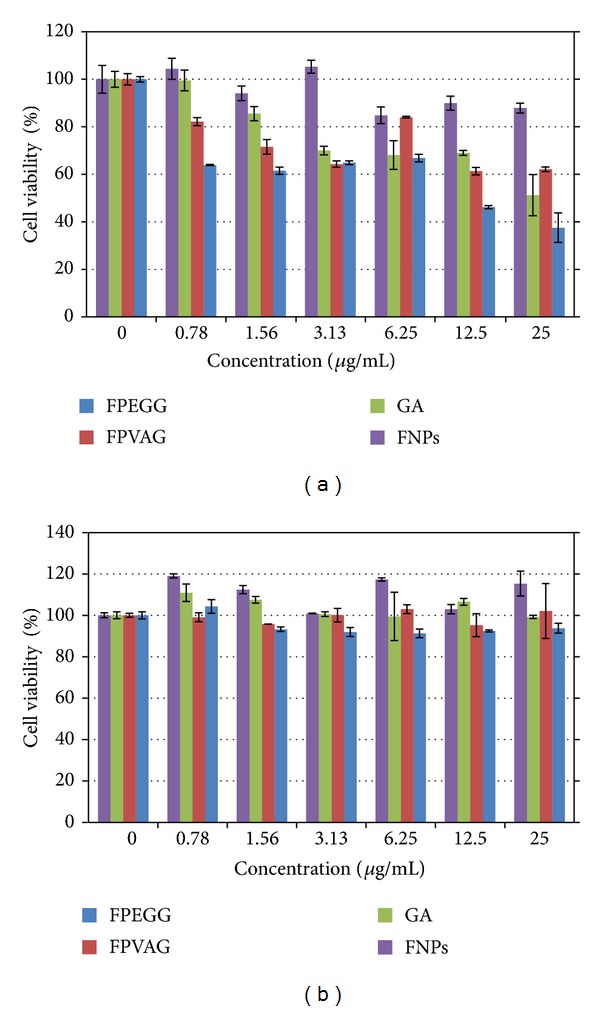
Cell viability assays of (a) MCF-7 cell lines and (b) MRC-5 cells and anticancer activity of FNPs, GA, FPEGG, and FPVAG nanocomposites, respectively, after 72 hours of treatment. FNPs: iron oxide nanoparticles; GA: pure gallic acid; FPEGG: iron oxide coated with polyethylene glycol and gallic acid; FPVAG: iron oxide coated with polyvinyl alcohol and gallic acid.

Normal lung cell and breast cancer cell lines were used to study the possible toxicity and anticancer effectiveness of the two nanocomposites as well as bare iron oxide and pure gallic acid in a dose-dependent manner.  Figure 8(a) shows dose-dependent effect of iron oxide nanoparticles, FPEGG and FPVAG nanocomposites, on MCF-7 cells. It also shows the effect of these nanocomposites compared to pure gallic acid on the same cells. Statistically, there is a significant difference between the FNPs-treated group and the FPEGG-treated group with *P* < 0.05 as tested by an ANOVA and a Turkey post hoc test. The IC_50_ values are 11.61 ± 0.12 **μ**g/mL, 16.63 ± 0.21 **μ**g/mL, 18.61 ± 0.37 **μ**g/mL, and 38.36 ± 0.16 **μ**g/mL for FPEGG, FPVAG, GA, and FNPs, respectively.Figure 8(b) demonstrated the viability study of MRC-5 cells following exposure to FPEGG and FPVAG nanocomposites as compared to FNPs and pure gallic acid after 72 hr, using increasing concentrations of each compound.

The viability was found to be maintained above 80% when normal lung cells were exposed to the iron oxide nanoparticles, pure gallic acid, and both nanocomposites (FPEGG and FPVAG) within the tested concentrations. A dose-dependent decrease in cell viability was seen following exposure to the same concentration of the two nanocomposites and pure gallic acid on a breast cancer cell line. Figures 8(a) and 8(b) show that the sustained cell viability is above 80% when both cancer and normal cell lines were exposed to increased concentration of empty iron oxide nanoparticles over 72 hr period. Interestingly, this indicates that the possibility of toxicity and/or anticancer effect on the cells could be due to the release of gallic acid from the nanocomposites and not owing to the empty iron oxide nanoparticles.

It was found that the FPEGG demonstrated higher anticancer effect on the breast cancer cell lines in almost all concentrations tested compared to FPVAG. There are less than 40% viable cells at 25 **μ**g/mL of FPEGG, while FPVAG has about 60% viable cells and pure gallic acid about 50% viable cells at 25 **μ**g/mL concentration (Figure 8(a)). Thus, in the above tested cell, FPEGG nanocomposite was found to have higher toxicity effect compared to both FPVAG and pure gallic acid. The uptake and retention of the nanoparticle is likely enhanced with a PEG coating than the PVA. This leads to different viability of the cancer cell when the same concentration of gallic acid in both FPEGG and FPVAG nanocomposites were used in this study. Previous study showed that the nanoparticles coated with vitamin E succinated polyethylene glycol 1000 (TPGS) were shown to have 1.4-fold increase in uptake compared to PVA coated nanoparticles [45].

## 5. Conclusion

The synthesized superparamagnetic iron oxide nanoparticles coated with polyethylene glycol-gallic acid and polyvinyl alcohol-gallic acid can be prepared by the coprecipitation method. Iron oxide nanoparticles have the mean size of 9 nm, compared to 31 nm and 35 nm for FPEGG and FPVAG nanocomposites. The coating process in both nanocomposites was found to improve the thermal stability of the two resulting nanocomposites compared to their uncoated counterparts. Although the release of the gallic acid from the two nanocomposites (FPEGG and FPVAG) was found to be of controlled manner through an anion exchange process, from the results it was found that the release profiles of GA from FPEGG were more sustained compared to the one from FPVAG nanocomposite. *In vitro* bioassay study showed that the FPEGG nanocomposite demonstrated higher anticancer effect on the breast cancer cell lines in almost all concentrations tested compared to FPVAG nanocomposite.

## Figures and Tables

**Table 1:** Magnetic properties of FNPs, FPEGG, and FPVAG nanocomposites.

Samples	Ms (emu/g)	Mr (emu/g)	Hc (G)
Fe_3_O_4_	64.655	1.5714	21.955
FPEGG	40.005	1.1334	14.727
FPVAG	38.635	0.8359	24.977

**Table 2:** Correlation coefficient, rate constant, and half-time obtained by fitting the data of the release of GA from FPEGG and FPVAG nanocomposites into phosphate buffered solutions at pH 4.8 and 7.4.

Aqueous solution	Saturated release %	*R* ^2^			Rate constant (*k*)^a^ (mg/min)	*t*_1/2_^a^ (min)
		Pseudo-first order	Pseudo-second order	Parabolic diffusion		
pH 7.4*	60.9	0.7893	0.9992	0.8651	6.41 × 10^−5^	254
pH 4.8*	89.4	0.9168	0.9950	0.9567	1.92 × 10^−5^	568
pH 7.4**	80.9	0.5984	0.9953	0.9816	3.49 × 10^−5^	323
pH 4.8**	86.4	0.9887	0.9989	0.8220	8.19 × 10^−5^	141

*Estimation was done for the release of GA from FPEGG nanocomposite. **Estimation was done for the release of GA from FPVAG nanocomposite and ^a^estimated using pseudo-second-order kinetics.
